# Changes in the Arctic Ocean Carbon Cycle With Diminishing Ice Cover

**DOI:** 10.1029/2020GL088051

**Published:** 2020-06-13

**Authors:** Michael DeGrandpre, Wiley Evans, Mary‐Louise Timmermans, Richard Krishfield, Bill Williams, Michael Steele

**Affiliations:** ^1^ Department of Chemistry and Biochemistry University of Montana Missoula MT USA; ^2^ Hakai Institute Heriot Bay British Columbia Canada; ^3^ Department of Earth and Planetary Sciences Yale University New Haven CT USA; ^4^ Woods Hole Oceanographic Institution Woods Hole MA USA; ^5^ Institute of Ocean Sciences Sidney British Columbia Canada; ^6^ Applied Physics Laboratory University of Washington Seattle WA USA

**Keywords:** Arctic Ocean, ice concentration, seawater CO_2_, interannual variability, Canada Basin, shipboard CO_2_ measurements

## Abstract

Less than three decades ago only a small fraction of the Arctic Ocean (AO) was ice free and then only for short periods. The ice cover kept sea surface *p*CO_2_ at levels lower relative to other ocean basins that have been exposed year round to ever increasing atmospheric levels. In this study, we evaluate sea surface *p*CO_2_ measurements collected over a 6‐year period along a fixed cruise track in the Canada Basin. The measurements show that mean *p*CO_2_ levels are significantly higher during low ice years. The *p*CO_2_ increase is likely driven by ocean surface heating and uptake of atmospheric CO_2_ with large interannual variability in the contributions of these processes. These findings suggest that increased ice‐free periods will further increase sea surface *p*CO_2_, reducing the Canada Basin's current role as a net sink of atmospheric CO_2_.

## Key Points

- The Arctic Ocean is losing ice cover at a rapid rate
- The loss of ice cover is leading to heating and uptake of atmospheric CO_2_, increasing sea surface CO_2_ levels
- Continued increase of sea surface CO_2_ will likely reduce the uptake of atmospheric CO_2_ in the future

## 1 Introduction

The rapid loss of sea ice in the Arctic Ocean (AO) is well documented (Meier et al., 2014). Other changes in the AO are also becoming evident. Freshwater content is increasing due to sea ice melt and river runoff (e.g., Krishfield et al., 2014; Proshutinsky et al., 2009; Yamamoto‐Kawai et al., 2009). Sea surface temperature has also increased (e.g., Perovich et al., 2011; Steele et al., 2008 *;* Timmermans*,* 2015; Toole et al., 2010). This evolving physical environment is altering biological production (Arrigo & van Dijken, 2015; Bergeron & Tremblay, 2014) and food web structure (Hunt et al., 2014; Li et al., 2009; Søreide et al., 2010). The carbon cycle in the AO is intimately connected to these processes (Anderson & Macdonald, 2015), but it is not clear how carbon sources and sinks are changing in the AO and if they could affect CO_2_ accumulation in the atmosphere and sea surface.

Sea surface *p*CO_2_ is a key carbon cycle parameter because it is used to determine air‐sea CO_2_ fluxes for global carbon budgets and for understanding the rate of ocean acidification. Despite this, sea surface *p*CO_2_ measurements in the AO are spatially and temporally sparse. While *p*CO_2_ measurements date back decades (Kelley*,* 1970) and have continued on sporadic research cruises (Ahmed et al., 2019; Bates et al., 2006; Cai et al., 2010; Evans et al., 2015; Jutterström & Anderson, 2010; Robbins et al., 2013), measurements are mostly from AO shelf regions in the summer and fall due to limited access to more heavily ice‐covered regions. Few studies have included repeat shipboard *p*CO_2_ measurements from the AO's deep basins. The interior basins comprise ~50% of the surface area of the AO (Bates et al., 2011) and, because of their reduced seasonal variability compared to AO coastal margins, might provide an earlier indicator of changes in *p*CO_2_ as the ice‐free sea surface is exposed to present‐day atmospheric CO_2_ levels.

While the AO is known to be a sink for atmospheric CO_2_ (Arrigo et al., 2010; Bates et al., 2011; Islam et al., 2016, 2017; Yasunaka et al., 2016, 2018), its contribution to global air‐sea CO_2_ fluxes remains highly uncertain (Anderson & Macdonald, 2015). Estimated to uptake between 70 and 200 teragrams (Tg) carbon per year or 5–14% of the global uptake (Bates et al., 2011; Yasunaka et al., 2018), continued ice loss will make these estimates even more uncertain. Previous studies have found evidence that *p*CO_2_ levels are changing in the oligotrophic AO basins and along the Chukchi shelf (Cai et al., 2010; Miller et al., 2014; Yasunaka et al., 2016, 2018), and a new analysis by Ouyang et al. (2020) more clearly shows rising levels of *p*CO_2_ in the Canada Basin since 1994. Increased CO_2_ uptake by the AO will also accelerate ocean acidification, that is, driving a commensurate decrease in pH that increases calcium carbonate solubility (Robbins et al., 2013; Yamamoto‐Kawai et al., 2009). In this manuscript, a shipboard *p*CO_2_ time series collected from 2012–2017 in the AO's Canada Basin reveals that *p*CO_2_ increases with decreasing ice concentration. We use a temporal reference, days since ice retreat (DSR), and a mass balance model to examine to what extent ice‐dependent processes such as air‐sea CO_2_ fluxes, surface ocean warming, and biological production drive changes in sea surface *p*CO_2_ after ice retreats.

## 2 Methods

### 2.1 Observations

Underway sea surface *p*CO_2_ was measured on the Beaufort Gyre Observing System/Joint Ocean Ice Study (BGOS/JOIS) cruises on the CCGS Louis S. St‐Laurent during 2012–2017. No *p*CO_2_ measurements were made in 2015. The starting dates for the five ~4 week cruises were 6 August 2012, 3 August 2013, 25 September 2014, 24 September 2016, and 8 September 2017. The *p*CO_2_ was recorded using an infrared‐gas equilibrator system (SUPER‐CO_2_, Sunburst Sensors, LLC) located in the ship's lab. The instrument uses an infrared analyzer (LI‐COR, LI‐840A) and gas phase equilibrator (Liqui‐Cel membrane contactor, Model #G453) as described in Hales et al. (2004). The equilibrator was connected directly to the ship's seawater line. Calibrations were automated using CO_2_ gas standards and a zero CO_2_ gas sample. Temperature was measured in the equilibrator and at the seawater intake (9 m depth), assumed equal to sea surface temperature (SST), as discussed below. The infrared analyzer CO_2_ mole fraction was corrected to SST and converted to *p*CO_2_ using 100% humidity at SST and local barometric pressure (Dickson et al., 2007). Some warming, usually <0.5°C, can occur enroute to the equilibrator so it is essential to correct the *p*CO_2_ for this temperature change. If there were greater than ~2°C differences between the equilibrator inlet temperature and SST, it was assumed seawater flow had stopped (e.g., due to ice clogging) and the *p*CO_2_ was discarded during these periods. A flow meter was installed in 2016 to detect periods of low flow rate. The *p*CO_2_ uncertainty is estimated to be ±5 μatm based on the reproducibility of the standards and baseline zero. CTD stations showed that the seawater intake was sometimes within the halocline (i.e., below the mixed layer), and this was also evident from the salinity and temperature variability recorded by the ship's thermosalinograph. These conditions were found mostly on the continental shelf during 2012 and 2013 due to high Mackenzie River outflow. This analysis focuses on the Canada Basin bounded by 155–130°W and 72–82°N where CTD stations consistently found that mixed layer depths were greater than the ship intake depth.

Air temperature, wind speed, wind direction, and barometric pressure were recorded by the ship's weather system. Mixed layer depths, defined as the depth where the density difference from the surface first exceeds 0.25 kg m^−3^ (Timmermans et al., 2012), were determined using temperature and salinity from ~50 CTD casts occupied annually as part of the BGOS/JOIS cruises (Proshutinsky et al., 2019). Atmospheric *p*CO_2_ was computed from the mole fraction of CO_2_ measured at Alert, Nunavut, Canada, using data from the National Oceanic and Atmospheric Administration (NOAA) Earth System Research Laboratory (ESRL) (https://www.esrl.noaa.gov). Sea ice concentration with daily, 12 km resolution was obtained from the French Research Institute for Exploration of the Sea (IFREMER) (http://cersat.ifremer.fr/oceanography-fromspace/our-domains-of-research/sea-ice) that provides data collected by the satellite‐based Special Sensor Microwave Imager (SSM/I) and processed by the National Snow and Ice Data Center (https://nsidc.org).

### 2.2 Data Analysis and Modeling

In this study our goals are to determine if sea surface *p*CO_2_ levels are related to interannual variability in ice concentration and to evaluate processes that might control *p*CO_2_ under low (or no) ice conditions. To facilitate analysis of the spatially and temporally disparate shipboard *p*CO_2_ and other data, Canada Basin data were gridded by identifying 20 × 20 km grid areas that contain data, and those data were then averaged as described in Evans et al. (2015). Underway *p*CO_2_ measurements collected at different times within each grid cell were averaged, and an average time of the measurements was computed. The ship might spend as much as a day within a 20 × 20 km area because of variable activities, for example, mooring deployments, so these underway data were averaged together to avoid a spatial bias. The gridding routine also computed the standard deviation of data found within each grid cell as well as the number of observations. The average number of observations for each grid cell ranged from 17–23 for each cruise. As a temporal reference relative to the beginning of the open water period, we define “days since ice retreat” or DSR, as the difference between the *p*CO_2_ measurement date and the day of ice retreat (DOR) for each grid cell. DOR is the day when ice concentration dropped below 15% in any grid cell (Steele & Dickinson, 2016), corresponding to the approximate uncertainty in ice concentration (Ivanova et al., 2015). The gridded data were also averaged together for each cruise (i.e., each year) to allow interannual comparisons of the mean physical and biogeochemical conditions. Further, we used these yearly values as input to the mass balance model described below.

The mass balance model was used to examine how sea surface *p*CO_2_ might change after ice retreats. Many processes contribute to sea surface *p*CO_2_ variability in polar regions including biological production, heating and cooling, physical mixing and upwelling, ice melt and formation, and air‐sea gas exchange. Sea surface warming and air‐sea uptake are likely the most important factors for increasing *p*CO_2_ in low ice areas in the Canada Basin (Cai et al., 2010; Else et al., 2013). The combined contributions of these two processes to sea surface *p*CO_2_ variability were estimated using a dissolved inorganic carbon (DIC) mixed layer mass balance (Islam et al., 2017; Martz et al., 2009) as follows:

(1) $$\Delta \mathrm{DIC}=F_{\text{gasex}}\times \Delta t/\left(\mathrm{MLD}\times \rho \right),$$

where Δ*DIC* is the change in DIC for a time step Δ*t* (1 hr in this study), *F*_gasex_ is the air‐sea CO_2_ flux (e.g., in mmol m^−2^ day^−1^), MLD is the mixed layer depth, and ρ is seawater density. Warming (increasing SST) is accounted for in the equilibrium calculation as described below. *F*_gasex_ was calculated using

(2) $$F_{\text{gasex}}=k\times K_{0}\times \Delta p{\mathrm{CO}}_{2}\times f,$$

where *k* is the gas transfer velocity, *K*_0_ is the CO_2_ solubility, Δ*p*CO_2_ is the *p*CO_2_ difference between the sea surface and atmosphere, and *f* is the fraction of open water (Butterworth & Miller, 2016; Prytherch et al., 2017). In this case, *f* is set equal to 1 because the *p*CO_2_ was modeled only after the day of ice retreat (DOR, defined above). *F*_gasex_ is negative when there is a net uptake of CO_2_ by the ocean from the atmosphere. We used the wind speed relationship in Wanninkhof (2014) to compute *k*, where ship wind speed was corrected to 10 m height. The average of second moments of wind speed (i.e., wind speed^2^) was calculated as opposed to wind speed averages because short‐term (<daily) variability in the winds is retained leading to higher gas transfer rates during periods of greater wind speed variability (Evans et al., 2015; Wanninkhof*,* 2014).

A simple modification of Equation 1 makes it possible to examine potential contributions from net community production (NCP),

(3) $$\Delta \mathrm{DIC}=\left(F_{\text{gasex}}+F_{\mathrm{NCP}}\right)\times \Delta t/\left(\mathrm{MLD}\times \rho \right),$$

where *F*_NCP_ is the net uptake of CO_2_ due to biological production (e.g., in mmol m^−2^ day^−1^). We used values from Ji et al. (2019) who measured NCP using O_2_ isotope and O_2_/argon methods during the same BGOS cruises, excluding 2017. In the mass balance models, DIC was incremented for each time step with Δ*DIC* from Equation 1 (gas exchange only) or Equation 3 (gas exchange and biological production). The *p*CO_2_ was then recalculated using the new DIC, SST, and salinity and a constant total alkalinity (A_T_) in the CO_2_ equilibrium program CO2sys (Pierrot et al., 2006). The A_T_ was estimated using an A_T_‐salinity relationship derived from bottle samples during the BGOS cruises (DeGrandpre et al., 2019; Yamamoto‐Kawai et al., 2005). A_T_ is a conservative property of seawater that does not change with temperature or air‐sea CO_2_ exchange (Millero*,* 2007). The time period of the model calculation is based on the maximum DSR for each cruise. For surface warming, SST was incremented equally for each time step from −1.5°C to the mean SST (Table 1) from DSR = 0 days until the end of the DSR period similar to Else et al. (2013). The initial under‐ice condition was chosen to be the freezing point of seawater (−1.5°C) at a salinity of 27 and a seawater *p*CO_2_ of 300 μatm all derived from extrapolation of the mean gridded data versus mean ice concentration to 100% ice concentration. All equilibrium (CO2sys) calculations used the Mehrbach et al. (1973) constants refit by Dickson and Millero (1987). Lastly, mean gridded values were used in the model calculations (Table 1 and Equations 1–3). A model that uses the evolution of ice concentration at each grid cell and includes other changing physical conditions, e.g. MLD or wind, was beyond the scope of this study.

**Table 1:** Mean Cruise Values Used in the Mass Balance Model Derived From Ship Measurements and Other Sources (See Section 2)

Year	Mean *p*CO_2_ (μatm)	Sea ice concentration (%)	Wind speed (m s^−1^)	Atm. *p*CO_2_ (μatm)	SST (°C)	Salinity	Mixed layer depth (m)
2012	365 ± 34	8 ± 22	8.1 ± 1.1	379 ± 3	2.5 ± 3.6	25.5 ± 1.4	12.1 ± 6.6
2013	327 ± 24	59 ± 38	5.0 ± 1.0	384 ± 3	−0.1 ± 1.6	26.7 ± 0.8	15.5 ± 5.5
2014	318 ± 14	78 ± 32	5.8 ± 0.7	392 ± 4	0.6 ± 2.2	27.0 ± 0.7	27.2 ± 4.3
2016	371 ± 23	21 ± 37	6.6 ± 0.3	395 ± 3	−0.3 ± 1.0	27.1 ± 0.7	26.1 ± 4.7
2017	350 ± 21	19 ± 33	7.3 ± 0.7	395 ± 2	1.1 ± 1.9	26.9 ± 1.0	26.5 ± 5.1

## 3 Results and Discussion

**Figure 1:**
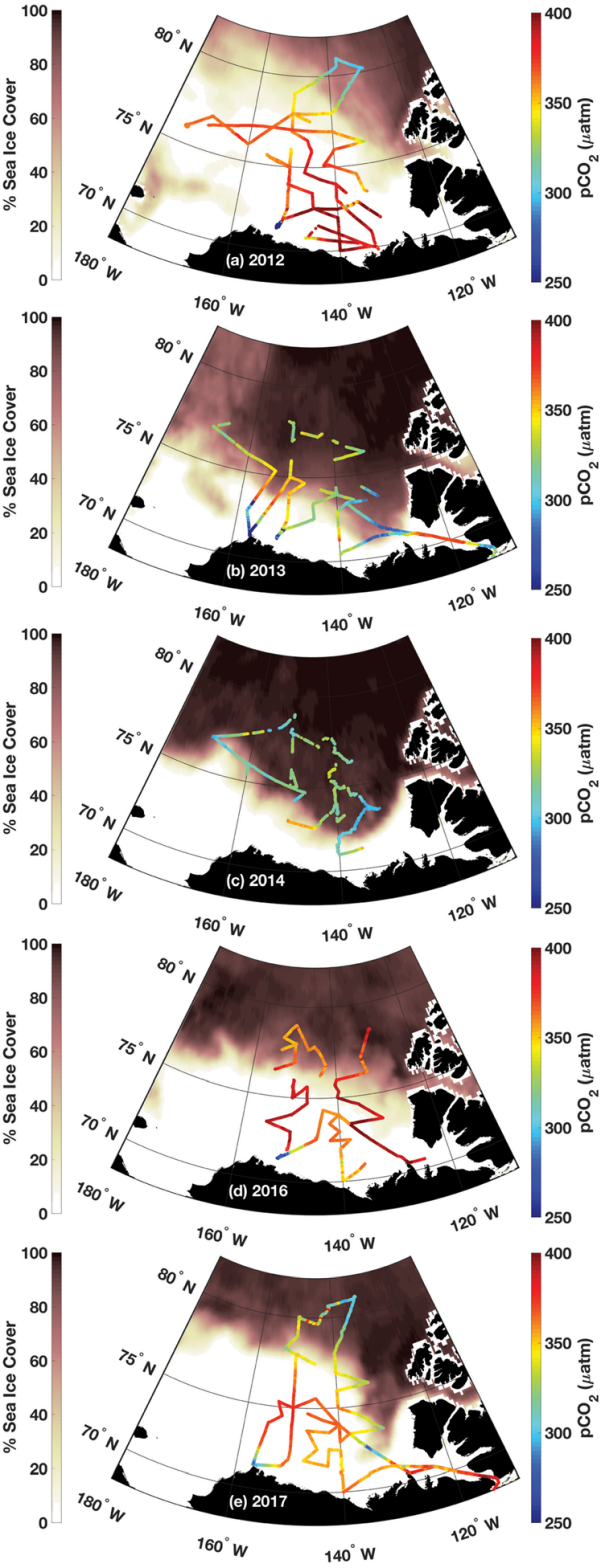
Sea surface partial pressure of CO_2_ (*p*CO_2_) data obtained on the Canadian icebreaker CCGS Louis S. St‐Laurent from 2012–2017. The *p*CO_2_ levels are indicated by the color along the ship cruise track (right color bar). The dark shaded coloration (left color bar) represents sea ice concentration averaged from the daily satellite data collected over the course of each cruise. Data for this analysis were taken from the area bracketed by 155–130°W, 72–82°N in the Canada Basin. The ship visited the same stations each year, but the cruise track varied to support other field studies and various other activities. The data gaps in 2013 were due to problems with the seawater intake. The starting dates for the five ~4 week cruises were 6 August 2012, 3 August 2013, 25 September 2014, 24 September 2016, and 8 September 2017, top to bottom, respectively. No *p*CO_2_ measurements were made in 2015.

The shipboard *p*CO_2_ data collected on the Beaufort Shelf, Canada Basin, and eastern Chukchi Sea are shown in Figure 1 overlaid on % sea ice concentration. A large range of interannual variability in sea ice cover was observed during these cruises. In 2012, ice cover dropped to 3.4 million km^2^, the lowest level observed since the satellite record began in 1978. The minimum ice extent rebounded in 2013 and 2014 to ~5.0 million km^2^. All 5 years ranked in the top 10 lowest minima (National Snow and Ice Data Center, Arctic Sea Ice News and Analysis, http://nsidc.org/arcticseaicenews). Sea surface *p*CO_2_ was also highly variable spatially and interannually. Open water in the Canada Basin typically had higher *p*CO_2_ levels than *p*CO_2_ recorded in ice‐covered areas; for example, compare 2012 (low ice) and 2014 (more ice) in Figure 1.

**Figure 2:**
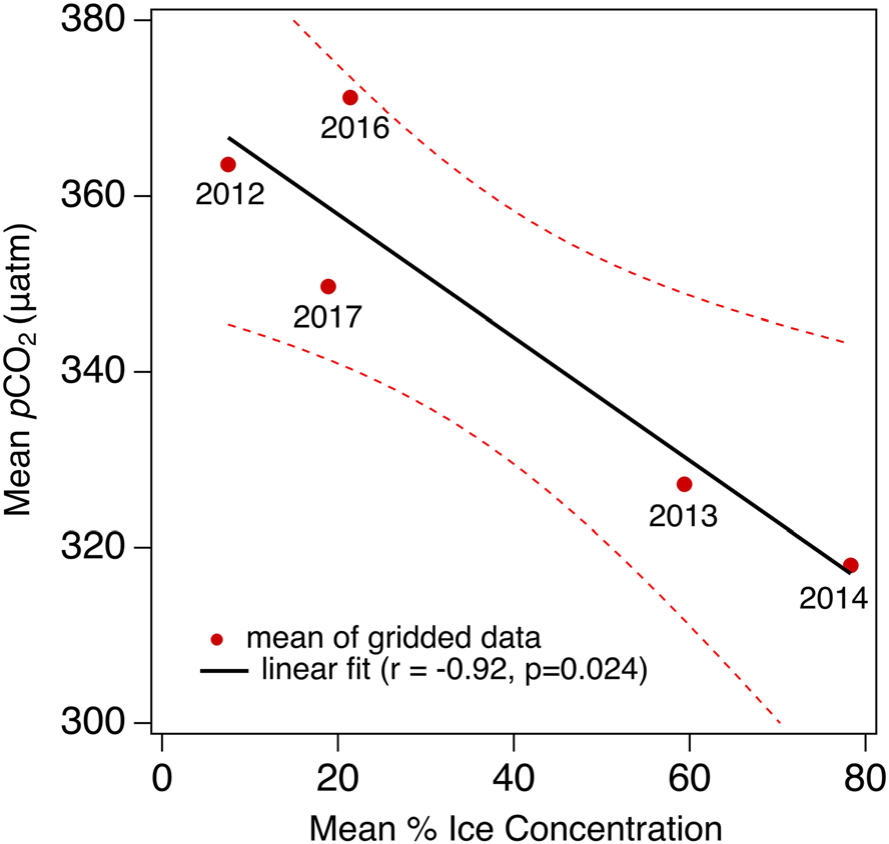
Canada Basin mean *p*CO_2_ versus mean sea ice concentration for each cruise shown in Figure 1. The slope of the least squares fit is −0.70 μatm/%. Symbols are labeled with each year. Means were computed using data gridded to a 20 × 20 km area in the region spanning 155–130°W and 72–82°N (Figure S1). The 95% confidence bands are included (dashed red curves). No measurements were made in 2015.

Using the mea levels for each cruise reveals a significant correlation with ice concentration (Figure 2). Sea surface *p*CO_2_ is higher and closer to atmospheric saturation during years of low ice concentration and exceeded atmospheric *p*CO_2_ (Table 1) in some locations in 2012 and 2016 (Figure 1). Although sparser data sets over different regions have found evidence that sea surface *p*CO_2_ in the AO is increasing with decreasing ice cover (Cai et al., 2010; Else et al., 2013; Jutterström & Anderson, 2010) and a new compilation of data since 1994 shows an increase in *p*CO_2_ in the Canada Basin (Ouyang et al., 2020), no previous studies have documented an interannual connection with ice concentration like that shown in Figure 2. The mean *p*CO_2_ values from each cruise reveal this relationship with ice concentration by consolidating the variability—the gridded *p*CO_2_ data for each cruise (shown in supporting information Figure S1) cover the full range shown in Figure 2 obscuring interannual differences.

The absence of sea ice exposes the surface ocean to direct solar radiation and atmospheric heating and the subsequent warming increases *p*CO_2_. Air‐sea exchange will also increase *p*CO_2_ because, when the *p*CO_2_ is lower than atmospheric levels, the AO will absorb CO_2_ from the atmosphere (Figure 1 and Table 1). These mechanisms for increasing *p*CO_2_ in the surface ocean suggest that *p*CO_2_ is not only dependent upon the ice cover but also the duration of open water (Arrigo & van Dijken, 2015). Thus, the days since ice retreat (DSR) are used as a temporal reference, as defined above. We examine specific variables that might be important in driving the relationship between *p*CO_2_ and sea ice concentration, including DSR, sea surface temperature (SST), MLD, wind speed, and NCP (Equations 1–3 and Table 1).

**Figure 3:**
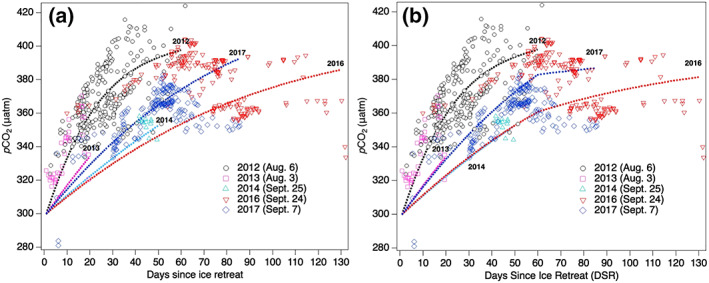
Gridded *p*CO_2_ observations (symbols) versus days since ice retreat (DSR) from 2012–2017, excluding 2015. Modeled *p*CO_2_ (dashed curves, labeled with each year and with the color matching the *p*CO_2_ symbol data) were computed from the predicted change in *p*CO_2_ due to air‐sea exchange and increase in SST using Equation 1 and values in Table 1. Models were run to the maximum DSR recorded for each cruise period with panel (a) using a constant heating rate and panel (b) heating only until DSR = 60, then temperature is held constant. Initial *p*CO_2_ before loss of ice was assumed to be 300 μatm (see section 2). The cruise start dates are indicated in parentheses in the legend.

The gridded sea surface *p*CO_2_ shows mostly increasing values after ice retreat with large intra and interannual variability (Figure 3a). Each year's observations appear to have a relatively consistent upward trajectory, except for 2016 and 2017 (discussed below), suggesting similar processes were at work in the open water area of the Canada Basin during each cruise. The *p*CO_2_ observations have more scatter with increasing DSR, possibly due to different physical conditions during each cruise and each year. Some of the interannual variability may be due to differences in cruise timing, but there are significant contrasts among cruises conducted over similar periods. For example, in Figure 2, the mean *p*CO_2_ from the August cruises (2012 and 2013) differs significantly, as do data from the late September cruises (2014 and 2017). These differences are also evident in the *p*CO_2_ data in Figure 3a with lower values in 2013 and 2014 compared to 2012 and 2016, respectively.

The results of the mass balance model (Equation 1) assuming constant heating are shown in Figure 3a. The model encompasses the range of observed variability using the mean conditions in Table 1. Although there is disagreement between observations and model curves during some years and some parts of the records, these results suggest that heating and/or gas exchange significantly contribute to the observed increase in *p*CO_2_ with increasing DSR and that these processes are highly variable from year to year (broken down in Figure S3). In 2012, the model predicts that heating and gas exchange increased *p*CO_2_ by ~60 and ~30 μatm, respectively; whereas in 2016, heating and gas exchange contributions were ~15 and ~65 μatm, respectively (Figure S3). Also note that a smaller range of variability is observed and predicted for the years with shorter DSR periods (2013 and 2014) (Figures 3 and S3), where surface ocean warming and air‐sea gas exchange were limited by the time of exposure to the atmosphere.

There are several other possible sources of variability and incorrect model assumptions that could contribute to differences between the model and observations. The sensitivity analysis in the Supporting Information shows that much of the variability within each cruise can be explained by varying input values over the observed ranges (Figure S2 and Table 1). There are some other notable deviations, however. For example, during 2016 and 2017, the 2 years with extended DSRs, the *p*CO_2_ levels decreased after ~60 days (Figure 3). In these years, data were collected into October, at which point sea surface cooling is possible which would decrease the *p*CO_2_. Because the model employs a linear warming trend, it can only predict the mean change not the time‐varying rate of warming or cooling. In Figure 3b, the model was modified so that all of the heating occurred before DSR = 60, slightly steepening the *p*CO_2_ increase but not markedly improving the prediction. We did not introduce an arbitrary period of cooling because this would essentially be fitting to the data. There is also evidence that movement of the ship into different water masses over the course of each cruise may have contributed to the variability. For example, the drop in *p*CO_2_ after 60 days in 2017 corresponds to a period of lower salinity (not shown). Also, employing DSR as a time variable in the model assumes that no air‐sea exchange or warming occurred when sea ice concentration was greater than 15% which is clearly not true (Figure S1). While we could not readily model gas exchange prior to DOR in this study, the heating contribution was small because no SST data exceeded 0.0°C from 0 to 10 days after ice retreat. An increase of −1.5°C, the approximate freezing point, to 0.0°C would increase *p*CO_2_ by ~22 μatm. Steele and Dickinson (2016) also found that SST is typically <0°C at DOR. Ice melt and formation could change *p*CO_2_ by diluting and concentrating DIC, respectively, and by altering the ratio of DIC and A_T_ in the ice or brine (Cai et al., 2010; DeGrandpre et al., 2019; Rysgaard et al., 2007, 2009). Ice melt occurs before DOR and could decrease *p*CO_2_ levels (Cai et al., 2010). In fact, heating, gas exchange, and ice melt may have all played a role in determining the pre‐DOR *p*CO_2_ levels, contributing to the deviations between model and observations evident in Figure 3.

Lastly, we consider possible biological contributions to the observed variability. Biological drawdown of sea surface *p*CO_2_ in the Canada Basin is predicted to be small because of the lack of nutrients in the stratified surface layer. The study by Cai et al. (2010) found no evidence of a biological DIC drawdown in the Canada Basin for waters >72°N. In a previous study in the Canada Basin, an NCP of ~4.9 mmol O_2_ m^−2^ day^−1^ offset a ~15 μatm *p*CO_2_ increase expected from atmospheric CO_2_ uptake in low ice conditions (Islam et al., 2017). The Ji et al. (2019) NCP values, which ranged from 1.3 to 2.9 mmol O_2_ m^−2^ day^−1^ from 2011–2016, confirm the very low productivity for the Canada Basin (Bates et al., 2005). For comparison, in the same paper, NCP in the eastern Chukchi Sea was estimated to range from 30 to 240 mmol m^−2^ day^−1^. The Canada Basin rates have a relatively small effect on the modeled *p*CO_2_ levels, estimated using Equation 3 (Figure S2). In 2012, the *p*CO_2_ would be reduced by 8–25 μatm by the end of the DSR period (60 days) over the range of NCP reported in Ji et al. (2019). These estimates assume the rate of NCP was constant over the DSR period. The O_2_/argon method integrates NCP over the residence time of O_2_ in the mixed layer (10–30 days) (Kaiser et al., 2005) and the rates in Ji et al. (2019) varied by only 15–21% during the cruises. This range of variability would not significantly alter the modeled *p*CO_2_ trajectory (Figure S2 and Table S1).

## 4 Conclusions

This study reveals that loss of sea ice leads to large interannual changes in sea surface *p*CO_2_ levels in the Canada Basin. Using the DSR as a temporal reference facilitated implementation of a time‐dependent mass balance model to explore the underlying mechanisms that might control sea surface *p*CO_2_ in open water conditions. Results from the model suggest that warming and air‐sea uptake drive the *p*CO_2_ toward atmospheric equilibrium to a varying extent (Figures 3 and S3). NCP is persistently low and can only account for a small portion of the observed interannual variability (Figure S2 and Table S1). These results suggest that neither of two previously proposed scenarios, that is, that increased SST will largely negate uptake of atmospheric CO_2_ (Else et al., 2013) or that air‐sea gas exchange, will consistently dominate the increase in *p*CO_2_ (Bates et al., 2006; Cai et al., 2010) (Figure S2). An important implication, as discussed by others (Cai et al., 2010), is that increased SSTs can reduce the uptake of atmospheric CO_2_ by increasing the rate at which *p*CO_2_ rises toward atmospheric equilibrium, decreasing the air‐sea CO_2_ gradient. The model simulation suggests that warming reduced the possible air‐sea CO_2_ flux by 55% in 2012, the year with the most warming (Figure S3). As Arctic warming and open water periods increase, it is possible that, as observed in 2012, the lowest ice concentration year on record, *p*CO_2_ will more frequently exceed atmospheric saturation. Over the period covered in this study any accumulation of CO_2_ is hidden by the interannual variability of ice cover, but the study by Ouyang et al. (2020) over a 23 year period clearly reveals this expected CO_2_ increase in the Canada Basin. Even with the insights provided in these new data sets, the future response of the AO carbon cycle to decreased seasonal ice cover remains highly uncertain and can only be understood by continued observations and modeling.

## Data Availability Statement

The data are available through the U.S. National Science Foundation (NSF) Arctic Data Center (https://arcticdata.io) (doi:10.18739/A2R785P3X).

## Supporting information



Supporting Information S1
