# Supplementary material for: Changes in the Arctic Ocean Carbon Cycle With Diminishing Ice Cover
Source: Geophys Res Lett. 2020 Jun 13;47(12):e2020GL088051. doi: 10.1029/2020GL088051 (PMC7380310; doi:10.1029/2020GL088051)
Supplement: Supplementary file 1 — Supporting Information S1 [file GRL-47-e2020GL088051-s001.docx]

**Supplemental Information**


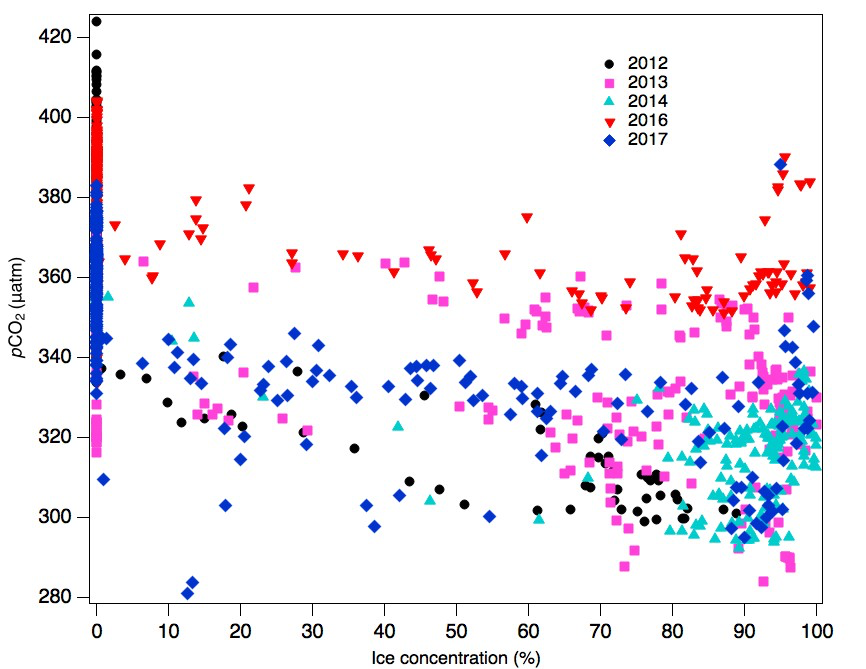
**Additional data:** All of the gridded *p*CO_2_ and ice concentration data from the maps (Figure 1) are plotted together in Figure 1S. These data reveal an overall upward trend of *p*CO_2_ with decreasing ice cover. Interannual differences are not clear, however. Consequently, each cruise data set was averaged to obtain Figure 2. The data after DOR, i.e. the Figure 1S data from 0-15% ice concentration, are the data that are plotted in Figure 3.

Figure 1S: Gridded *p*CO_2_ and ice concentration data for the area bracketed by 155-130° W, 72-82° N in the Canada Basin (Figure 1).

**Mass balance model sensitivity:** Model sensitivity to different input variables is illustrated by comparisons with the 2012 model from Figure 3A using 2012 average conditions (black curves in Figure 2S). Model inputs were varied over the range expected based on the inter-annual differences and standard deviations in Table 1 (MLD, wind speed and temperature) and the range of NCP in *Ji et al.* (2019). Results were similar for other years. The 2012 gridded *p*CO_2_ data are included as in Figure 3A to compare the full range of observed variability with the range predicted by the model. Each sensitivity run includes the contribution from heating so that sensitivity model curves and the 2012 model are directly comparable. These results show that much (but not all) of the variability observed during 2012 can be explained by variability in model input values, discussed in more detail in the manuscript. Sensitivities are shown in Table 1S, calculated from the relative standard deviation of the *p*CO_2_ divided by the relative standard deviation of the variable, i.e.,

S = [(sd *p*CO_2_ mean)/( *p*CO_2_ mean)]/[(sd variable)/[(variable mean)] Equation 1S

where S is the sensitivity in %/%, “sd *p*CO_2_ mean” is the average standard deviation of the model *p*CO_2_ from the 2012 Figure 3 curve (i.e., the deviations of the colored curves from the black curve) and “sd variable” is the standard deviation of the variable over the ranges shown in the legends in Figure 2S. The sensitivities (Table 1S) are small values because the range of *p*CO_2_ is large relative to the differences in *p*CO_2_, in contrast to the variable (e.g. wind) relative standard deviations which are large (the denominator). The numbers can be compared, however, to provide insight into the potential importance of each variable. To facilitate this comparison, the sensitivities are normalized to the sensitivity due to heating (Table 1S). Results are most sensitive to wind speed, reflecting the non-linear (quadratic) relationship between wind speed and gas transfer velocity (Wanninkhof 2014).

Figure 2S: Sensitivity of the model (see Methods) to different input values for 2012 including wind speed (A) mixed layer depth (B), net community production (NCP)(C) and heating (D). Values were varied over the ranges shown in Table 1. NCP was varied over the range measured by *Ji et al.,* (2019).


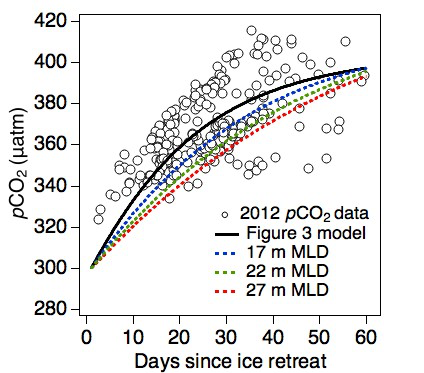

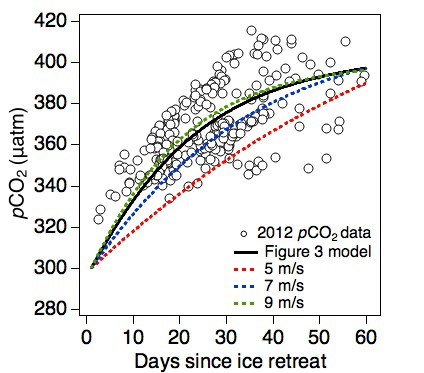

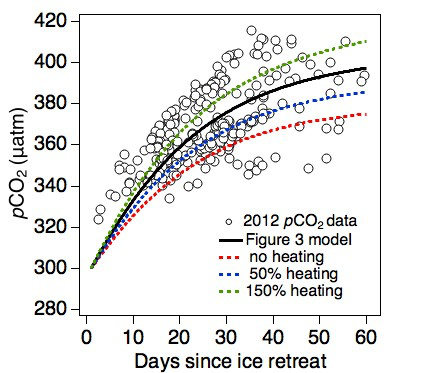

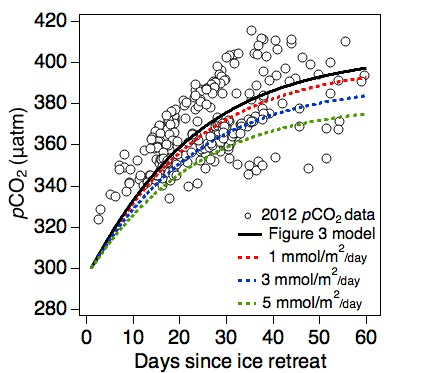


**A**

**B**

**C**

**D**

| Table 1S: Sensitivity analysis comparisons. | | |
| --- | --- | --- |
| **Sensitivity** | **%/%** | **relative to heating** |
| Heating | 0.037 | 1.00 |
| MLD | 0.047 | 1.27 |
| Wind speed | 0.090 | 2.43 |
| NCP | 0.025 | 0.68 |

Figure 3S: Modeled air-sea CO_2_ fluxes (left) and maximum modeled changes in sea surface *p*CO_2_ (right) for open water. The right figure was derived directly from the model results in Figure 3A using the mean values in Table 1 (no NCP). In the left panel, NCP was set equal to 1.32 mmol m^-2^ day^-1^ (*Ji et al.,* 2019) with and without the SST increase in Table 1.


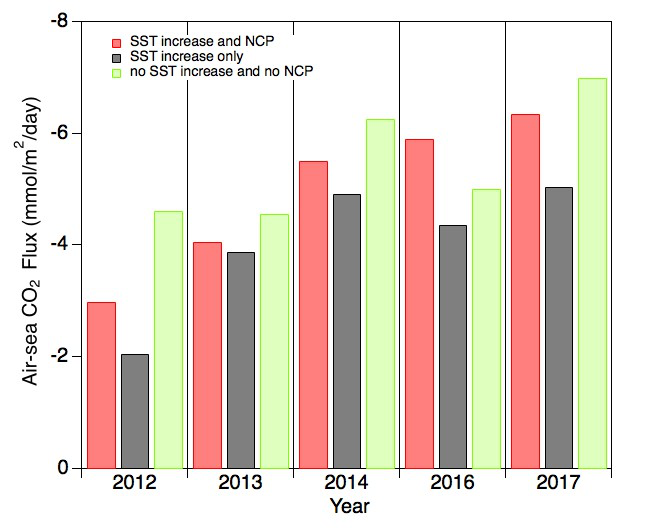

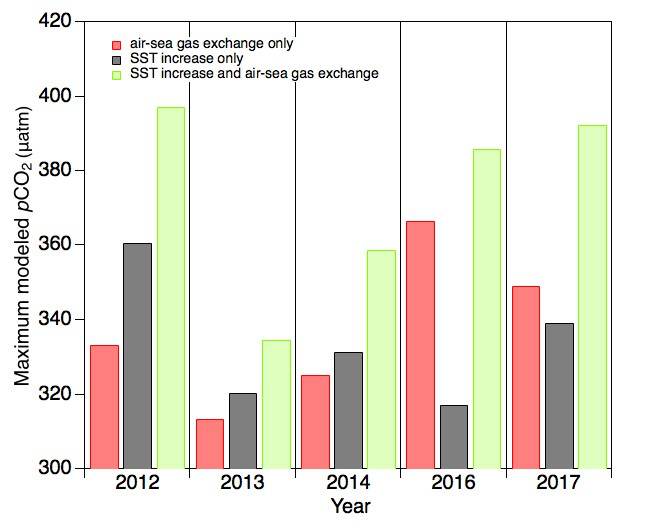


**Effects on air-sea CO_2_ fluxes and changes in *p*CO_2_**: The model is also used to assess the influence of the SST increase and NCP on the air-sea CO_2_ flux (Figure 3S, left panel). These calculations were determined from the model results shown in Figure 3A with SST warming only or with SST warming and NCP. The fluxes are comparable to other open water air-sea flux values reported for the AO (*Bates et al.*, 2011; *Evans et al.,* 2015). Both SST and NCP can change the net flux of CO_2_ by increasing and decreasing the rate of increase of *p*CO_2_, respectively. Fluxes are lowest (within years) when SST is included in the model due to the more rapid increase in *p*CO_2_ towards atmospheric equilibrium. The net air-sea CO_2_ flux is lowest in 2012 because the *p*CO_2_ exceeded atmospheric levels and became a source of CO_2_ for a short period (Figure 3). For this reason, the increase in SST had the strongest influence in 2012, decreasing the air-sea CO_2_ flux by 55% compared to 13-28% during the other years. The model shows that the mean NCP reported in *Ji et al.* (2019) (1.32 mmol m^-2^ d^-1^) slows the increase in *p*CO_2_, and consequently, increases the flux by 45% in 2012 and from 5-35% during the other years (Figure 3S, left panel). The predicted change in *p*CO_2_ was computed to further illustrate the influence of SST increase on sea surface *p*CO_2_ (Figure 3S, right panel). These results suggest that the relative contributions from the increase in temperature and air-sea gas exchange vary significantly from year to year. For example, contrast the changes in *p*CO_2_ for the larger increase in temperature and shorter maximum DSR in 2012 with the small increase in temperature and long DSR in 2016 (Table 1, Figure 3A). Consequently, these results suggest that under current conditions the Canada Basin sink for atmospheric CO_2_ will have large inter-annual variability.
